# The Molecular Basis of Glucose Galactose Malabsorption in a Large Swedish Pedigree

**DOI:** 10.1093/function/zqab040

**Published:** 2021-08-17

**Authors:** M Pilar Lostao, Donald D Loo, Olle Hernell, Gunnar Meeuwisse, Martin G Martin, Ernest M Wright

**Affiliations:** Departments of Physiology and Pediatrics, The David Geffen School of Medicine at UCLA, Los Angeles, CA 90095-1751, USA; Department of Physiology, The Geffen School of Medicine, UCLA, USA; Departments of Physiology and Pediatrics, The David Geffen School of Medicine at UCLA, Los Angeles, CA 90095-1751, USA; Departments of Physiology and Pediatrics, The David Geffen School of Medicine at UCLA, Los Angeles, CA 90095-1751, USA; Departments of Physiology and Pediatrics, The David Geffen School of Medicine at UCLA, Los Angeles, CA 90095-1751, USA; Departments of Physiology and Pediatrics, The David Geffen School of Medicine at UCLA, Los Angeles, CA 90095-1751, USA

**Keywords:** sodium glucose cotransporter, SGLT1, glucose-galactose malabsorption Swedish GGM pedigree, GGM mutation SGLT1 structure

## Abstract

Glucose-galactose malabsorption (GGM) is due to mutations in the gene coding for the intestinal sodium glucose cotransporter SGLT1 (SLC5A1). Here we identify the rare variant Gln457Arg (Q457R) in a large pedigree of patients in the Västerbotten County in Northern Sweden with the clinical phenotype of GGM. The functional effect of the Q457R mutation was determined in protein expressed in *Xenopus laevis* oocytes using biophysical and biochemical methods. The mutant failed to transport the specific SGLT1 sugar analog α-methyl-D-glucopyranoside (αMDG). Q457R SGLT1 was synthesized in amounts comparable to the wild-type (WT) transporter. SGLT1 charge measurements and freeze-fracture electron microscopy demonstrated that the mutant protein was inserted into the plasma membrane. Electrophysiological experiments, both steady-state and presteady-state, demonstrated that the mutant bound sugar with an affinity lower than the WT transporter. Together with our previous studies on Q457C and Q457E mutants, we established that the positive charge on Q457R prevented the translocation of sugar from the outward-facing to inward-facing conformation. This is contrary to other GGM cases where missense mutations caused defects in trafficking SGLT1 to the plasma membrane. Thirteen GGM patients are now added to the pedigree traced back to the late 17^th^ century. The frequency of the Q457R variant in Västerbotten County genomes, 0.0067, is higher than in the general Swedish population, 0.0015, and higher than the general European population, 0.000067. This explains the high number of GGM cases in this region of Sweden.

## Introduction

Next-generation sequencing has rapidly improved the ability to identify novel monogenic disorders, including congenital diarrheas and enteropathies (CODE) in young infants.^1^^,^^2^ Chronic CODE disorders are generally classified as either secretory diarrheas resulting from abnormal electrolyte transport or malabsorptive diarrheas caused by failure to absorb nutrients. Malabsorption may be generalized, caused by impaired assimilation of numerous nutrients associated with abnormalities of enteroendocrine function or epithelial trafficking and polarity abnormalities.^3^^,^^4^ In contrast, selective malabsorption is caused by abnormalities in the digestion or absorption of specific nutrients.

**Figure 1.**
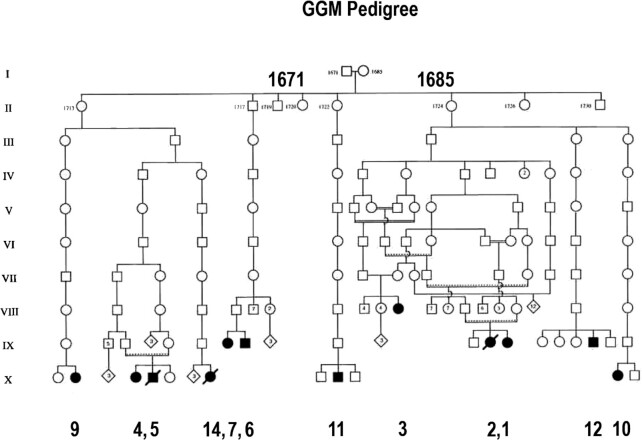
The Northern Swedish Pedigree of GGM patients. The original pedigree, containing cases 1–6, filled symbols (square male, circle female), were traced back to a couple in the late 17^th^ century.^7^^,^^8^ Four suspected cases were also identified in a family with 12 children (hatched symbol) of a distant relative to case 3. Six additional GGM patients: 7, 9–12, and 14, have now been placed on this pedigree, but DNA was only obtained for 6 identified cases in all: 1, 9–12, and 3 others not placed in the pedigree (15–17). The known GGM cases were traced to 4 of the 13 siblings born between 1719 and 1724.

The first CODE disorder described at both a clinical and molecular level was glucose-galactose malabsorption (GGM), a potentially lethal defect in intestinal sugar absorption, was independently reported in 1962.^5^^,^^6^ Subsequently, this clinical phenotype was identified in 6 cases within a pedigree located in Northern Sweden.^7^^,^^8^ A simple therapy, removing glucose, galactose, and lactose (and starch from older infants) from the diet, effectively treated these patients. We established that GGM is due to mutations in the gene coding for the intestinal brush border sodium glucose cotransporter SGLT1 (SLC5A1).^9–13^ In over 80 GGM patients, most mutations were missense, but nonsense, frameshift, splice-site, and promoter mutations were identified. The most common defect of Na^+^/glucose cotransport amongst the missense mutations is a failure to insert the transporter into the plasma membrane.^14^ We were intrigued about the cause of GGM in the Västerbotten pedigree traced back to the end of the 17^th^ century (Figure 1).^7^^,^^8^ We were fortunate to obtain a blood sample from the first case identified on the pedigree, 8 others diagnosed with the disorder and a few first-degree relatives. A common Q457R mutation was responsible for the defect in glucose and galactose transport, and subsequent studies of Q457C and Q457E mutants showed that the positive charge on Q457R is responsible. Examining whole-genome sequences of a control Västerbotten population revealed a higher frequency of the Q457C variant than in the general Swedish and European populations.

## Methods

### Cases

We focused on identifying the mutation(s) that caused GGM in the pedigree first identified by Melin and Meewuisse (1969) in northern Sweden (Figure 1).^7^^,^^8^ Amongst the 6 patients in this pedigree, we obtained a blood sample from case 1 for genetic analysis. Ultimately, we identified an additional 16 GGM cases referred to the Department of Pediatrics at the University of Umeå for diagnosis or consultation and blood samples from 9, including cases 1, 9, 10, 11, 12, 15, 16, and 17. In two cases, 9 and 11, duodenal biopsies were taken as part of their clinical care, but immunohistochemistry on patient 11 was unsuccessful for technical reasons. Patients were born between 1961 and 2004, except case 3, born in 1926 and related to cases 1 and 2. Meeuwisse and Melin (1969) reported on the diagnosis of patients 1–6.^7^^,^^8^

This research was conducted in accordance with ethical standards of University of California at Los Angeles, under the jurisdiction of the Chancellor's Human Research Committee and subject to the 1964 Declarations of Helsinki and its later amendments. Written informed consent was obtained from each subject, or the parents of each minor child, for the collection of a blood sample and for sharing intestinal biopsies collected for clinical diagnosis.

The newly diagnosed cases had symptoms in common with previous ones in that they presented with diarrhea within days after birth. Glucose was in their stools, and diarrhea ceased immediately when carbohydrates, apart from fructose, were removed from their diet. In addition, blood glucose levels remained flat following oral glucose tolerance tests (2 g/kg). In all cases, the diarrhea was controlled by reducing the carbohydrate content of their diet, initially by feeding an infant formula with fructose as the only carbohydrate. The parents of GGM patients were healthy, and those able to be tested had normal oral glucose tolerance tests. Interviews amongst relatives and examination of parish registers (available in the Umea City Library) were used to add new cases on the pedigree.

### Identification of the Mutation, Site-Directed Mutagenesis and cRNA Synthesis

Genomic DNA was extracted from the blood sample of patients 1, 9, 10, 11, 12, 15, 16, and 17, and single-stranded conformational polymorphism (SSCP) screened abnormalities in the *SGLT1* gene in each exon, and mutations identified by Sanger sequencing, as previously described.^9–12^ All patients had a mutation in exon 12, a homozygote mutation of glutamine 457 to arginine. In later cases, exon 12 of the *SGLT1* gene was polymerase chain reaction (PCR) amplified and Sanger sequenced. PCR site-directed mutagenesis was performed using wild-type (WT) human SGLT1 cDNA as a template.^9^^,^^10^ The sequence of the synthetic oligonucleotide used to produce the Q457R mutation in the sense orientation was 5′-CGATTACATCC**G**GTCCATCACCAGT-3′, where the bold letter represents the mutated nucleotide. The mutant plasmid was linearized with XbaI, and in vitro transcription was performed with MEGAscript^TM^ transcription kit from Ambion by standard methods.^9^^,^^12^

### Uptake and Electrophysiology Experiments on Xenopus Oocytes

Mature female *Xenopus laevis* were anesthetized with 0.1% tricaine (Sigma-Aldrich, St Louis, MO) buffered with 0.1% NaHCO_3_ to harvest a portion of the ovary. Stage V-VI oocytes were selected and maintained at 18°C in modified Barth's solution, supplemented with 50 mg l^−1^ gentamicin (Sigma), 5.75 mg l^−1^ ciprofloxacin (Bayer), and 100 mg l^−1^ streptomycin sulphate/100000 units l^−1^ penicillin G sodium (Invitrogen, Carlsbad, CA). One day after isolation, oocytes were injected with 50 ng of cRNA coding for WT hSGLT1 or mutants and incubated at 18°C for 4–7 days. Experiments were performed at 20–22°C. Noninjected (NI) oocytes from the same donor frog served as controls. All animal protocols followed guidelines approved by the University of California Chancellor's Committee on Animal Research and the ARRIVE (Animal Research: Reporting of In Vivo Experiments) guidelines 2.0.

Oocytes obtained from adult *X. laevis* were injected with 50 nl (1µg/µl) of cRNA coding for WT or Q457R mutant human SGLT1.^15–17^ After 3 days, 50 µM ^14^C-α-methyl-D-glucoside (αMDG) uptake was measured.^17^^,^^18^

SGLT1 membrane currents were measured in the oocytes expressing either WT or mutant Q457R proteins, using a 2-electrode voltage clamp method.^17^^,^^19^^,^^20^ The steady-state sugar-dependent currents were obtained for each membrane voltage as the difference between the current measured at steady state in the presence and absence of sugar in 100 mM Na^+^. At each voltage, the currents recorded at different sugar concentrations were used to obtain the transporter's apparent affinity (K_0.5_) for the sugar.^21^ The transient SGLT1 charge movements (Q), with each 100 ms voltage pulse from the holding potential of −50 mV to each test membrane potential V_m_ between +50 and −150 mV, were calculated by integrating the presteady-state current (current obtained at 100 mM Na^+^ in the absence of sugar). The data were fitted to the Boltzmann equation: (Q-Q_qhy_)/Q_max_ = 1/[1 + exp(V_m_-V_0.5_)zF/RT], where maximal charge Q_max_ = Q_dep_—Q_hyp_ (Q_dep_ and Q_hyp_ are the charges at depolarizing and hyperpolarizing limits), F is the Faraday constant, R is the gas constant, T is absolute temperature, V_0.5_ is midpoint voltage, and *z* is the apparent valence of the voltage sensor.^20^^,^^21^ The time constant (τ) of the SGLT1 transient currents (or presteady-state currents) at each membrane potential was estimated for the ON-currents when membrane potential was stepped to the test potential from the holding potential (−50 mV). The transient ON currents were fitted to a single exponential equation: I = I_o_ exp(-**t**/τ), where I is the transient current (total current after subtraction of the oocyte capacitive and steady-state current), I_o_ is the maximum current at the beginning of the pulse, and **t** is time after the onset of the voltage-step.^19^^,^^20^^,^^22^ All the experiments were performed at 22°C.

### Western Blot Analysis

The WT and Q457R mutant SGLT1 proteins were extracted from cRNA-injected oocytes as previously described.^11^^,^^12^^,^^23^ The volume equivalent of 1/3 of oocyte was run on a 12% sodium dodecyl-sulfate polyacrylamide gel electrophoresis (SDS-PAGE) mini-gel and electro-transferred to nitrocellulose membrane. As controls, NI oocytes extracts and rabbit brush border membrane vesicles samples were loaded in the same gel. SGLT1 was detected using an anti-peptide polyclonal antibody raised to residues 602–613 in the C-terminal portion of rabbit SGLT1 at a dilution of 1:1000.^12^

### Freeze-fracture and Immunohistochemistry

Oocytes expressing WT or Q457R mutant SGLT1 and NI oocytes were fixed and freeze-fractured as previously described.^11^^,^^24^^,^^25^

Small intestinal biopsies from GGM patient 9 (Figure 1) and a standard control subject were fixed for 2 hr in Carnoy's solution (60% absolute alcohol, 30% chloroform, 10% acetic acid) and embedded, sectioned, and immune-probed as previously described.^23^ Incubation with the primary antibody was performed overnight in an anti-peptide antibody raised to residues 564–575 of the rabbit SGLT1 amino acid sequence at 1:100 dilution.^26^ After washing with phosphate-buffered saline (PBS) 3 × 10 min, sections were covered with 10 µg/ml rhodamine-labeled affinity-purified goat anti-rabbit IgG (Jackson Immuno Research Laboratories, Inc.) for 40 min and then washed with PBS 3 × 10 min. Nuclei were further stained with 2mg/ml DAPI (4',6-Diamidino-2-phenylindole dihydrochroride, Research Organics Inc)-for 40 min. As controls, normal tissue sections were incubated with normal rabbit serum and labeled secondary antibody, or incubated with PBS but not with primary antibody before staining with the secondary antibody.

## Results

**Figure 2.**
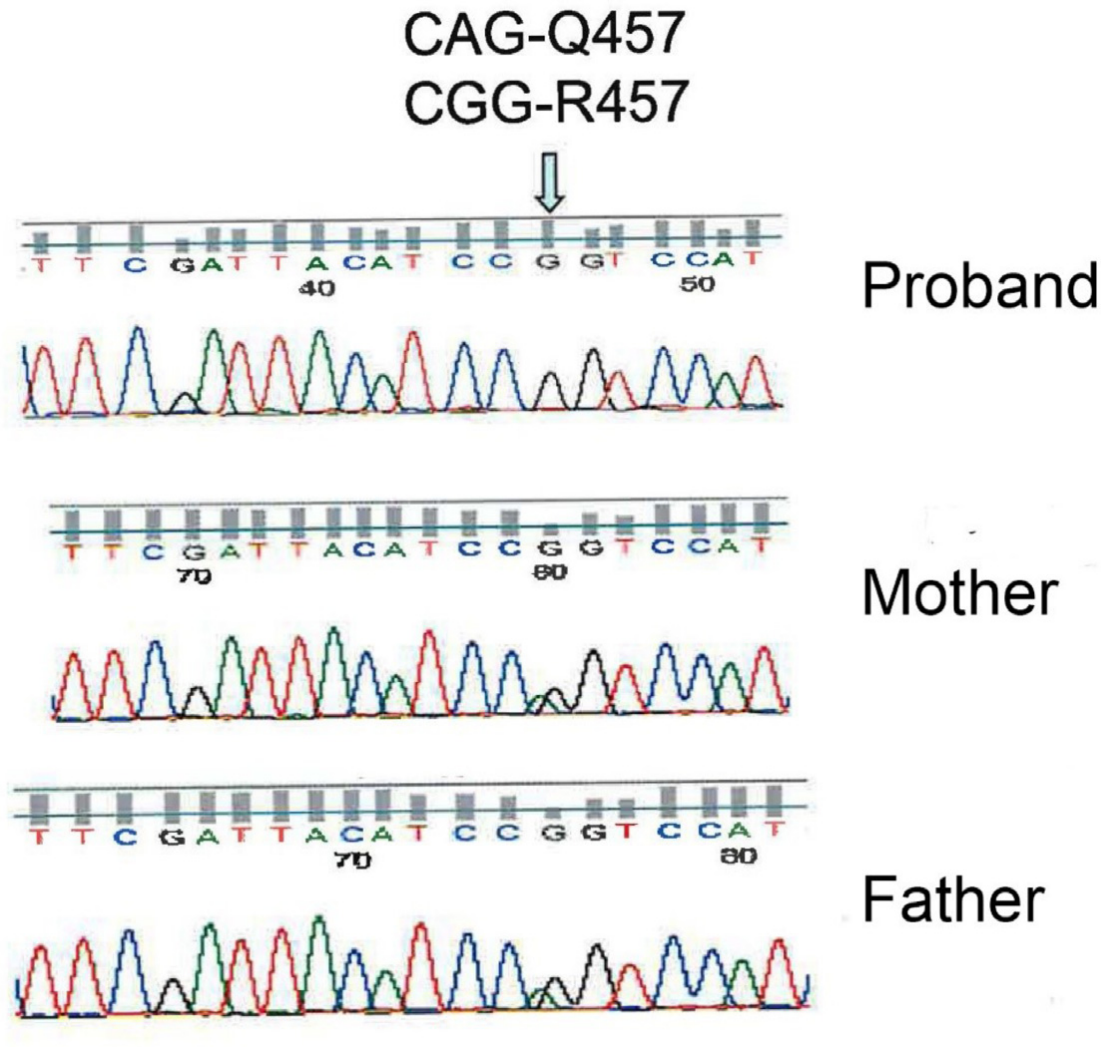
Sanger sequencing of child with GGM and his parents. The proband has a CGG-R457 mutation, while the mother and father are heterozygotes.

Molecular analysis of the *SGLT1* (*SLC5A1*) gene in 9 GGM patients (1, 9, 10, 11, 12, 15, 16, and 17) revealed a variant exon 12 by SSCP, and sequencing of the exon in both alleles showed a CAG to C*G*G mutation at nucleotide 1380 resulting in a Q457R mutation (Figure 2). Where genomic samples were available, parents of cases 9, 15, 16, and 17 were heterozygotes, and unaffected siblings of cases 12, 16, and 17 were either heterozygotes (12, 16) or normal (17). This confirms the autosomal recessive mode of inheritance previously noted.^7^^,^^8^^,^^27^

**Figure 3.**
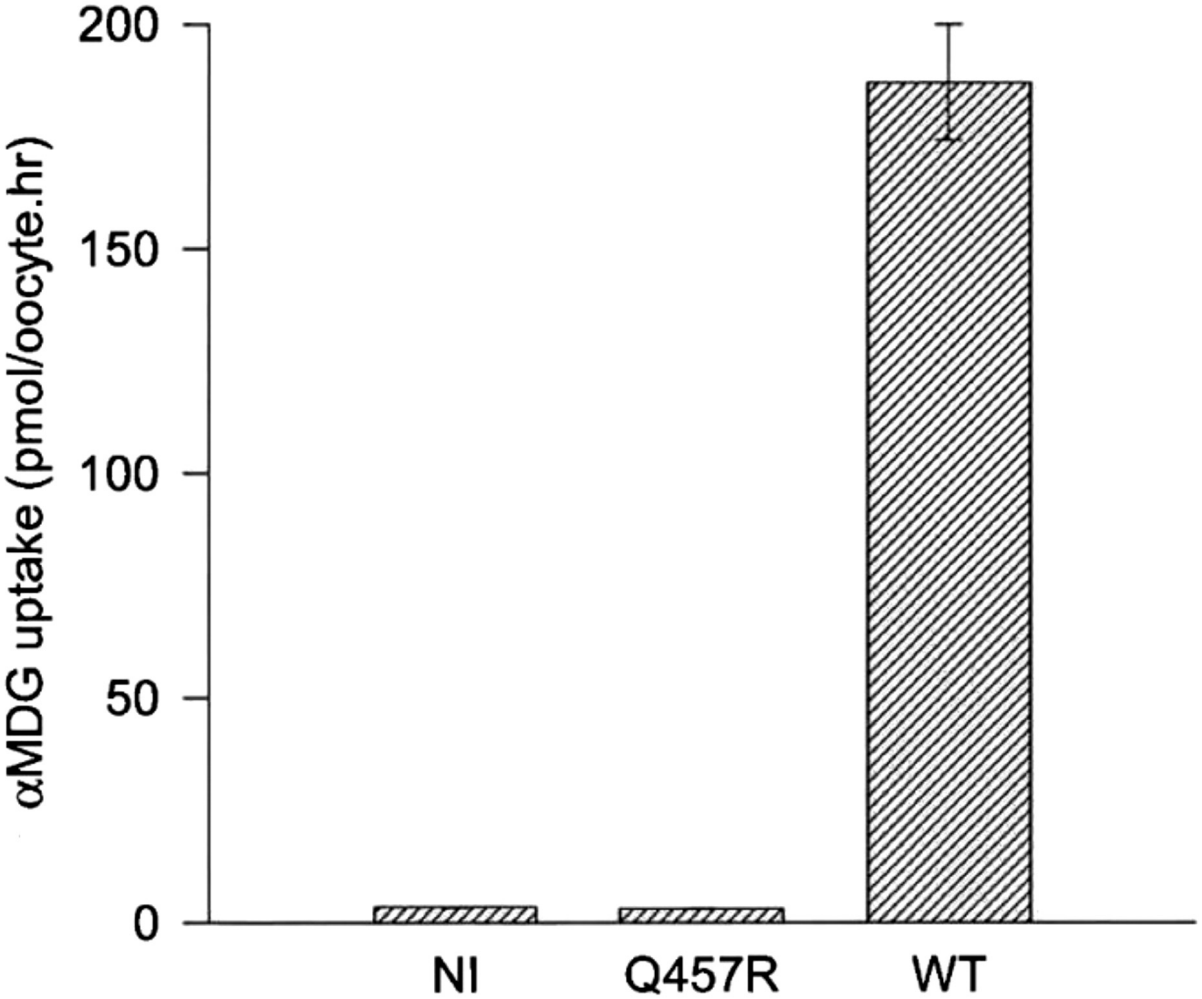
αMDG uptake by WT and Q457R mutant SGLT1. Oocytes were injected with 50 nl (1 µg/µl) of cRNA coding for WT or Q457R mutant SGLT1. After 3 days, uptake of 50 µM ^14^C-αMDG was measured. Values represent the mean of 8 oocytes and the error bars indicate the standard error. Q457R mutant uptake was not different from uptake by NI oocytes, 1.7% of the WT uptake (3 vs. 180 pmoles/h/oocyte).

**Figure 4.**
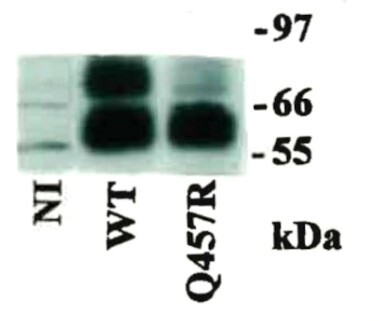
Western blot analysis of WT and Q457R mutant SGLT1 expressed in oocytes. Seven days after injection with WT or Q457R mutant cRNA, protein was extracted from 2 oocytes each. The equivalent to 1/3 oocyte was run in a 12% SDS-PAGE gel and after transfer to a nitrocellulose membrane, and was probed with an SGLT1 antipeptide antibody raised to residues 602–613, at 1:1000 dilution. Both WT and Q457R mutant proteins ran as 2 broad bands, one 70 kDa band which corresponds to the complex glycosylated form, less intense in the mutant, and another of ∼60 kDa which corresponds to core glycosylated form.^11^^,^^23^

**Figure 5.**
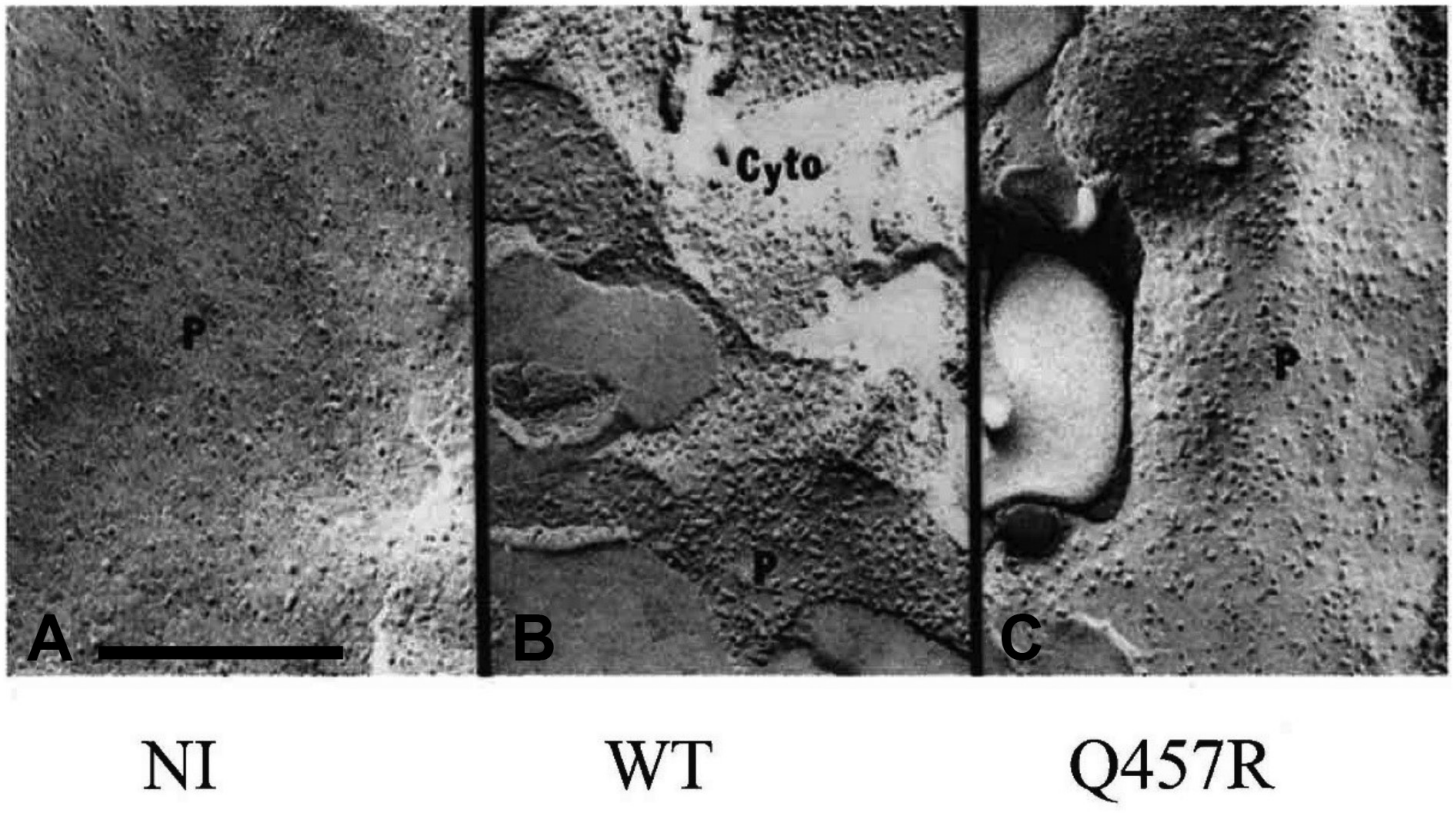
(Freeze-fracture) P fracture face of the plasma membrane of a NI oocyte and oocytes injected with Q457R or WT cRNA. NI oocyte (A) showed 7-nm-diameter particles in the P-face at a density of 200/mm^2^, whereas in WT injected oocyte (B), the density of particles increased to 1000/mm^2^. Similar density was obtained in Q457R cRNA injected oocytes (C). Q457R injected oocyte is the same as the one in Figures 7B and 8A. The 25 mM Na^+^/glucose current was −800 nA at –50 mV for the WT oocyte and 0 for Q457R (Figure 7); the SGLT1 charge movements were 8.5 and 3.7 nC (Figure 8A). Scale bar 200 nanometers.

To investigate if the Q457R mutant was functional, we measured the uptake of 50 µM ^14^C-αMDG by oocytes injected with WT and Q457R SGLT1 cRNA (Figure 3). Uptake by the mutant protein was 1.7% of WT uptake (180 pmoles/h/oocyte) and not different from NI oocytes (3.1 ± 0.1 and 3.4 ± 0.1 pmol/h/oocyte, respectively). These results indicate either that the mutant protein was not synthesized or that it was not trafficked correctly between the endoplasmic reticulum and the plasma membrane or functionally defective. To distinguish between these alternatives, we determined the expression of the mutant protein in oocytes using western blotting and the density of Q457R SGLT1 in the oocyte plasma membrane using freeze-fracture electron microscopy, and functional properties of Q457R using electrophysiology. Na^+^/glucose cotransport generates an inward Na^+^ current directly proportional to glucose transport, and SGLT1 in the plasma membrane exhibits charge movements Q that are directly proportional to the density of the protein in the membrane.^14^ Western blotting shows that the mutant protein was synthesized and glycosylated, as was the WT protein (Figure 4). The complex and core glycosylated Q457R protein pattern at 70 and 60 kD was similar for WT and mutant protein, even though the level of complex glycosylation was lighter for the mutant than WT. We have previously shown that N-linked glycosylation is not required for the functional expression of SGLT1.^27^ Freeze-fracture electron microscopy revealed that SGLT1 was inserted into the P-face of the plasma membrane of oocytes, and the density of the 7 Å particles was directly proportional to SGLT1 functional expression.^24^^,^^25^Figure 5 shows representative freeze-fracture images of NI oocytes and oocytes injected with WT and Q457R SGLT1 cRNA. All oocytes were processed in parallel from the same donor frog after measuring SGLT1 steady-state and pre-steady-state currents (see below). The density of the SGLT1 particles in the P-face of the control oocyte was 200/µm^2^, and this increased to 1000/µm^2^ for the oocyte injected with WT SGLT1 cRNA, and 600/µm^2^ for Q457R SGLT1. These results for control oocytes and those injected with human WT SGLT1 cRNA agreed with previous results.^12^^,^^25^ The density of Q457R in the plasma membrane was lower than for WT, and this is consistent with the lower level of functional expression of the mutant protein (on average, Q_max_ 3.7 nC for Q457R vs. 8.8 nC for WT, see below). These results suggest that the defect in αMDG uptake (Figure 3) is not due to defective mutant Q457C SGLT1 synthesis or trafficking from the ER (endoplasmic reticulum) to the plasma membrane but to an incompetent transporter.

**Figure 6.**
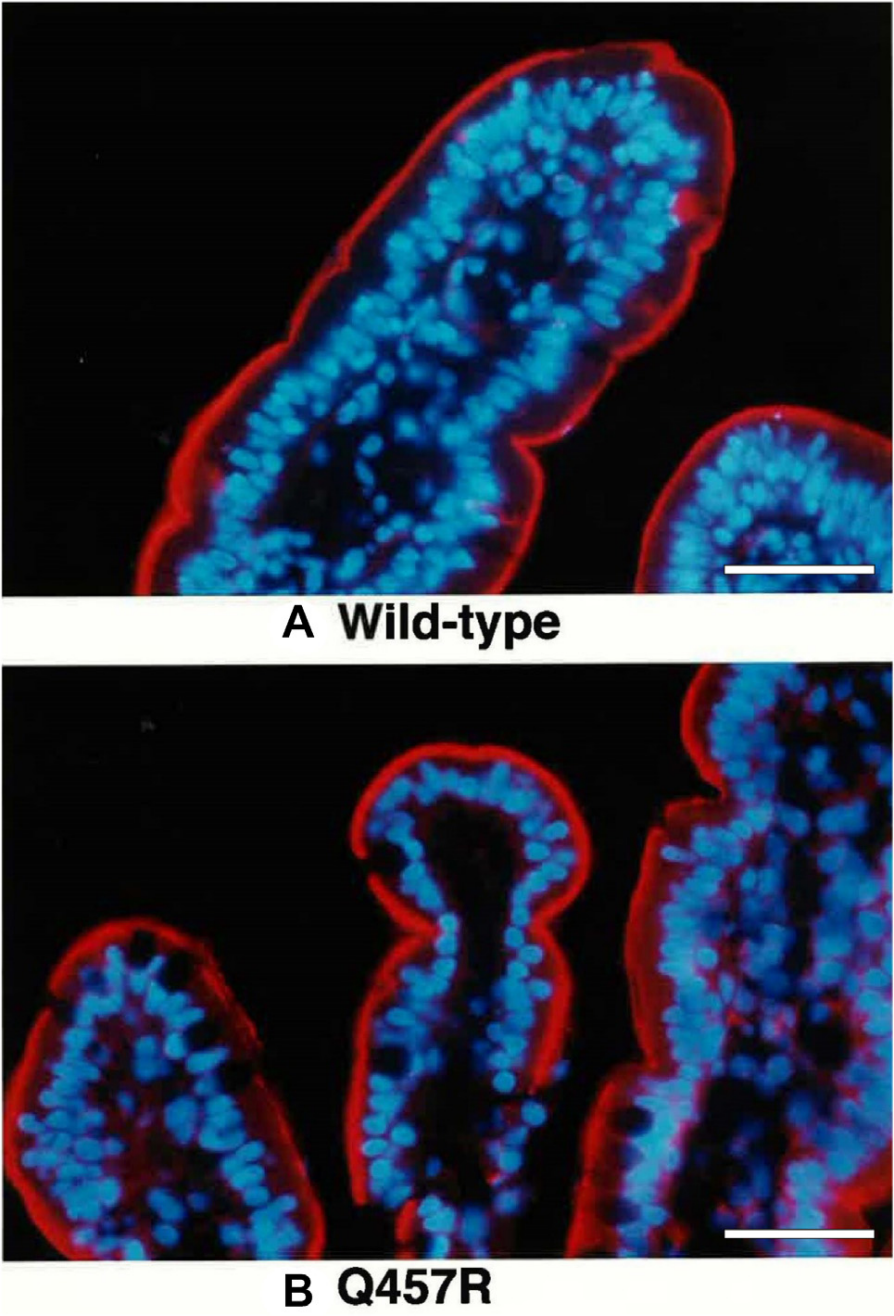
Immunolocalization of SGLT1 protein in human small intestine of a normal (control) (A) and a GGM patient (9) with the Q457R missense mutation (B). Double staining procedures shows SGLT1 (red) as a sharp line localized to the brush border of the enterocytes and the basal nucleus stained in blue. Goblet cells between the enterocytes were unstained for SGLT1. There were no differences in location of SGLT1 in enterocytes from the normal and the GGM (magnification x125). In a separate case of GGM, C292Y, the mutant protein did not reach the brush border membrane and was trapped just above the nuclei.^14^ Scale bar 30 micrometers.

Does the expression of mutant SGLT1 in the oocyte reflect the expression in the patient's enterocytes? Small bowel biopsies from GGM patient 9 was examined by immunohistochemistry (Figure 6). The mutant protein was detected as a thin red line over the brush border membrane of enterocytes, just as in a biopsy from a control subject. No immunofluorescence of SGLT1 protein was detected in control sections incubated with control rabbit serum. However, given the low resolution of light microscopy, the immunofluorescence does not actually show that the mutant SGLTs are inserted into the plasma membrane of the enterocyte brush border in the GGM patient, but only close to it.

**Figure 7.**
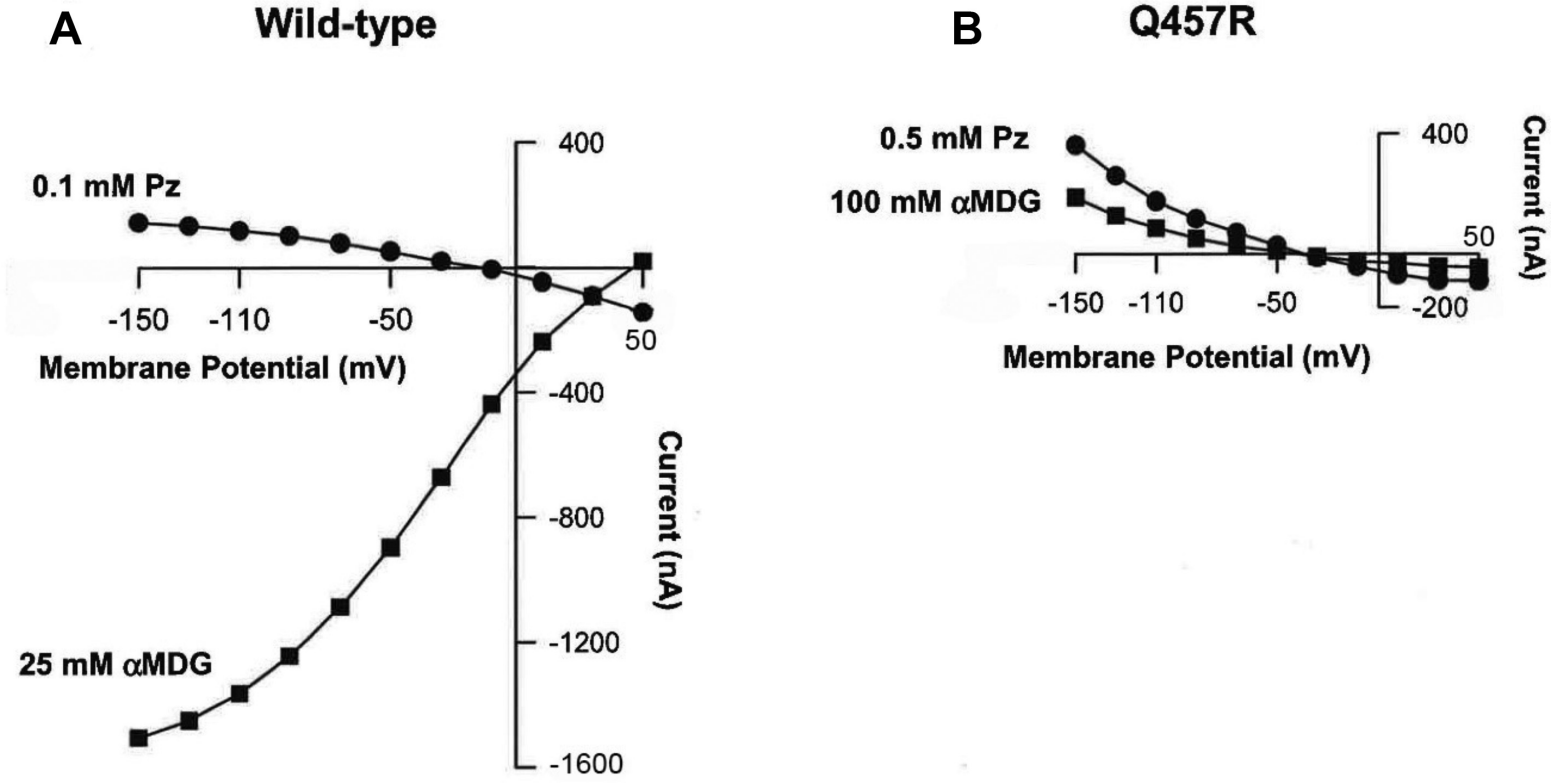
Steady-state currents induced by αMDG and phlorizin in WT and Q457R mutant proteins. Seven days after injection with cRNA, oocytes expressing WT and mutant SGLT1 were perfused with 100 mM NaCl buffer and currents were measured using a 2-electrode voltage clamp. The difference in steady-state currents measured in the absence and in the presence of αMDG or phlorizin (Pz) is plotted at each test potential from −150 to +50 mV. **A**. In WT, 25 mM αMDG induced Na^+^ inward current (1500 nA at −150 mV) whereas 0.1 mM Pz blocked the Na^+^ leak current (+143 nA at −150 mV). **B**. In Q457R mutant, both 100 mM αMDG and 0.5 mM Pz inhibited the Na^+^ leak current (+180 and + 360 nA at −150 mV, respectively). Similar results were obtained on oocytes from 3 different frog donors. The oocyte expressing Q457R-cRNA is the same one as shown in the oocyte in Figure 8B (Q/V with and without sugar) and Figure 5 (freeze-fracture).

Electrophysiological studies provided insight into the functional defects of Q457R SGLT1 in the plasma membrane. Figure 7 shows examples of the steady-state currents recorded in oocytes expressing the WT and Q457R proteins. The currents elicited by 25 mM αMDG and 0.1 mM phlorizin for WT SGLT1 depend on voltage: inward Na^+^ current increased in a sigmoid fashion from 0 nA at +50 mV to 1500 nA at −150 mV (Figure 7A). This current required the presence of external Na^+^ and was reversibly blocked by the addition of 0.1 mM phlorizin, the specific, non-transported competitive inhibitor (not shown). The Na^+^/glucose current is directly proportional to the rate of glucose transport, with a coupling of 2 Na^+^ and 1 glucose.^14^ The addition of 0.1 mM phlorizin in the absence of external glucose (closed circles) produced a small outward current that reached a value of 143 nA at −150 mV. This so-called SGLT1 leak current was not observed in NI oocytes.

In contrast, no inward sugar-stimulated Na^+^ currents were observed for the mutant even when the external αMDG concentration was raised to 100 mM, but instead, αMDG inhibited the Na^+^-leak current (Figure 7B). This leak current was blocked by 0.5 mM phlorizin and amounted to 360 nA at −150 mV. αMDG reduced the Q457R leak current in a concentration-dependent manner, with an apparent inhibitory constant K_i_ of 52 ± 4 mM, 2 orders of magnitude higher than the apparent K_m_ for Na^+^/glucose cotransport (0.34 ± 0.02 mM). The K_i_ for phlorizin, based on the concentration needed to inhibit the Q457R leak, was an order of magnitude higher than that for the WT leak. These results suggest that αMDG and phlorizin bind to Q457R, albeit at low affinities.

**Figure 8.**
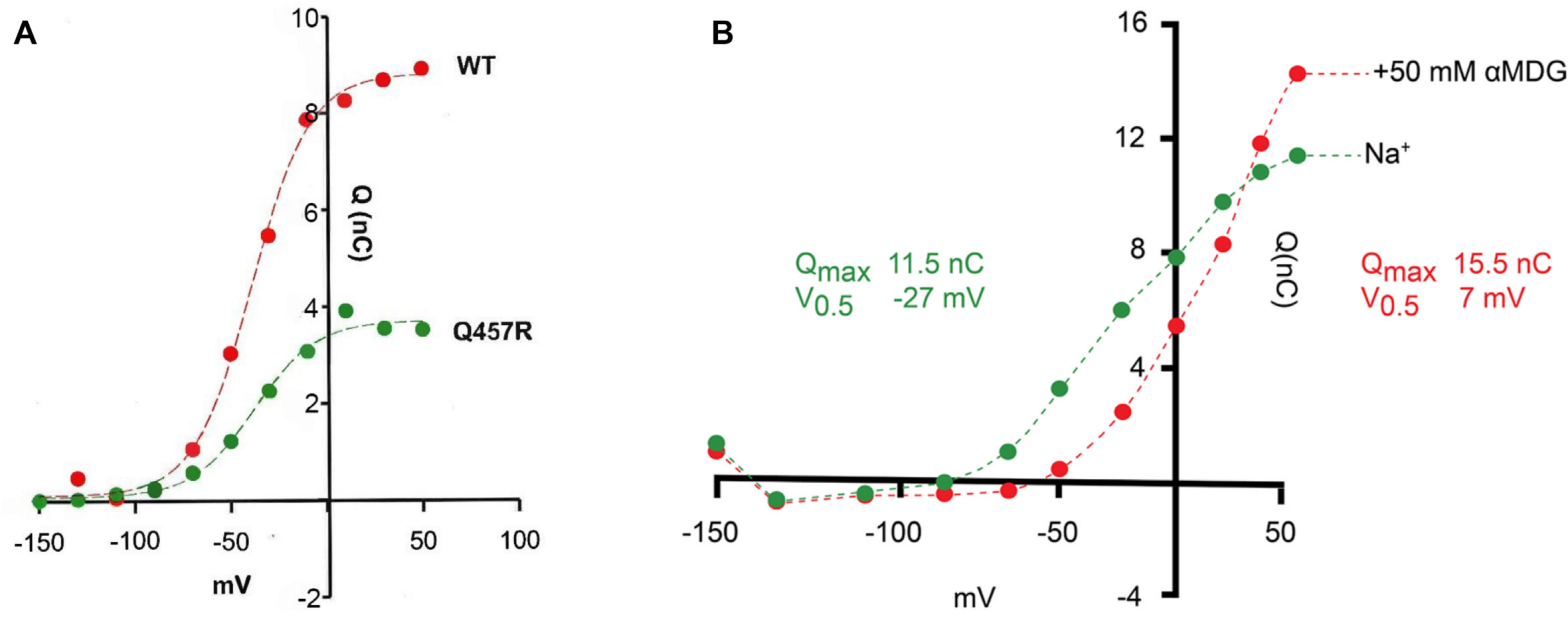
A. Charge-voltage (Q/V) relationships for WT and Q457R SGLT1. A. Charge was obtained by integration of SGLT1 current transients in the absence of sugar (see methods). The symbols correspond to the experimental data after the subtraction of the NI oocyte values, and normalized to WT charge at + 50 mV. The dotted lines are drawn according to the Boltzmann relation. Mutant Q_max_ (maximal charge transfer) was 40% of the WT value (3.7 ± 0.2 vs. 8.8 ± 0.3 nC). The V_0.5_ values were −47 and −50 mV, and *z* 1.4. B. Q/V relationships of Q457R SGLT1 in the absence and in the presence of the 50 mM αMDG. Q was obtained by the integration of current transients in the absence and in the presence of 50 mM αMDG. The symbols correspond to the experimental data and the curves are drawn according to the Boltzmann relation. The addition of 50 mM αMDG induced an increase of Q_max_, from 11.5 to 15.5 nC, and a shift of V_0.5_ from −27 to + 7 mV_,_ but no change in *z* (1.1).

In the presence of Na^+^ and absence of sugar, SGLT1 exhibits presteady-state currents after step changes in membrane potential. These reflect the conformational changes of the transporter in the membrane (charge movement, Q). Voltage-jump experiments showed that the mutant exhibited presteady-state currents characteristic of WT, indicating that it was in the plasma membrane. Figure 8 shows the charge/voltage relationship Q/V for WT and Q457R mutant transporters. The data were fitted to a Boltzmann relation, and the curves were similar except that Q_max_ for Q457R was only 60% on average of that for WT, 3.7 ± 0.2 nC vs. 8.8 ± 0.3 nC. Q_max_ is related to the number N of SGLTs in the plasma membrane: N = Q_max_/*ze*, where *z* is the apparent valence of the moveable charge (the limiting slope of the Q/V curve), and *e* is the elementary charge.^11^^,^^12^^,^^20^^,^^23^^,^^24^ There were no differences in the apparent valence (*z*) or the V_0.5_ (membrane potential for 50% maximal charge), which were 1.4 ± 0.1 and −46 ± 4 mV for the mutant, and 1.4 ± 0.1 and −50 ± 6 mV for WT. The 40% reduction in Q_max_ for the mutant relative to the WT agrees with the freeze-fracture electron micrographs shown in Figure 5.

In WT, the addition of external sugar blocked the presteady-state currents.^14^^,^^21^^,^^22^ Sugar reduced Q_max_ and shifted V_0.5_ to positive values with K_0.5_ values of 1 mM. Phlorizin also blocked Q_max_ with a K_i_ of 0.1 uM but with no shift in V_0.5_. In contrast, sugar did not abolish the presteady-state currents of Q457R but instead increased Q_max_ with a positive shift in V_0.5_ (Figure 8B). Q_max_, at 50 mM αMDG, was 15.5 nC relative to 11.5 nC in the absence of sugar. In this experiment, V_0.5_ shifted from −27 mV in the absence of sugar to +7 mV in the presence of 50 mM αMDG. There was no change in *z* (1.1). These sugar-induced changes in capacitive current were accompanied by a shift in the voltage dependence of the relaxation time constants. The time constant was independent of voltage between +50 and −100 mV at 4 ms but increased steeply between −100 and −150 mV to 40 ms, while the time constant for WT SGLT1 increased steadily between +50 and −150 mV from 4 to 28 ms.

## Discussion

In 1969, Meeuwisse and Melin identified 6 GGM patients in Northern Sweden, and another 4 suspected cases, who are pedigree members traced back to the 17^th^ century.^7^^,^^8^ We have extended this discovery with an additional 11 GGM cases and placed 6 on the pedigree (Figure 1). In addition, we have identified the mutation in the *SGLT1* (*SLC5A1*) gene responsible for the malabsorption syndrome, Q457R, in 8 cases (6, 9–12, 15–17). This variant is relatively frequent, 0.0067, in the genome of 300 Västerbotten residents over 80 years of age in the ACpop genomic database.^28^ This frequency is 4.5-times higher than a cross-section of the Swedish population, 0.0015, in the SweGen genomic database,^29^ and an order of magnitude higher than both the European (non-Finnish) and Finish genomes, 0.00011 and 0.00014, and 0.000067 in the GnomAD databases.^30^ The high frequency of the Q457R in Västerbotten genomes and the pedigree dating back to the 17^th^ century (Figure 1) is probably due to a founder effect, combined with the historic low population density and large distances between towns and villages.

The Q457R mutant expressed in oocytes is defective in Na^+^/glucose cotransport, as judged by the lack of αMDG-uptakes and absence of glucose-stimulated Na^+^ inward currents (Figures 3 and 7B). This defect is not due to the common trafficking defect as in other GGM mutants where the protein is trapped between the endoplasmic reticulum and the plasma membrane (D28N, L149R, C292Y, A304V, G318R, C355S, A468V, and R499H),^9^^,^^12^^,^^13^ but instead a defect in glucose transport across the plasma membrane. As evident from freeze-fracture electron microscopy (Figure 5), and current and charge movements (Figures 7 and 8A), Q457R is inserted into the plasma membrane and retains partial function. Namely, αMDG blocks the Q457R Na^+^-leak and binds to the transporter, shifting the Q/V curves (Figure 8B). Likely, the defective Q457R SGLT1 is also inserted into the brush border membrane of enterocytes in patients (Figure 6), unlike the C292Y GGM mutant that is trapped between the nuclei and brush border membrane.^14^

### Changes in SGLT1 Kinetic Model for WT and Mutant Q457R

**Figure 9.**
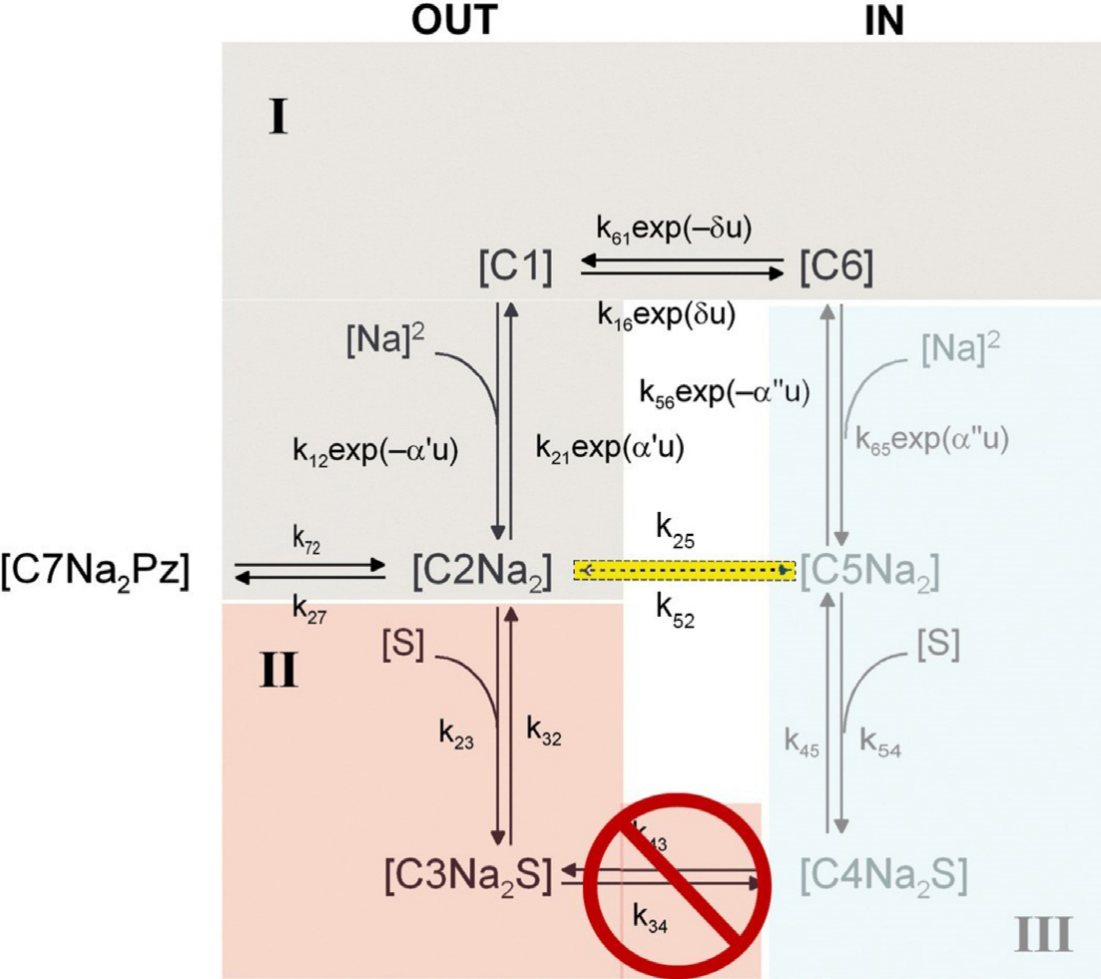
Six-state kinetic model of Na^+^/glucose uptake by SGLT1 expressed in oocytes. The external Na^+^ concentration was 100 m-equiv/l and external glucose concentration was 0–100 mM. As indicated in light blue the internal Na^+^ was 5 m-equiv/l and the cytoplasm was sugar-free. For simulations, the rate constants are: WT hSGLT1: k_12_  =  14000 M ^–2^s^–1^, k_21_ = 300 s^–1^, k_16_ = 600 s^–1^, k_61_ = 25 s^–1^, k_23_  =  45000 M ^–1^s^–1^, k_32_ = 20 s^–1^, k_34_ = 50 s^–1^, k_43_ = 50 s^–1^, k_45_ = 800 s^–1^, k_54_ = 7800 M^–1^s^–1^, k_65_ = 4500 M^–2^s^–1^, k_56_ = 10 s^–1^, and k_25_ = 0.3 s^–1^. k_54_ = 7800 M^–1^s^–1^ and k_52_ = 0.001 s^–1^ were determined by microscopic reversibility.^15^ The rate constants of mutant Q457R that accounted for its kinetics were the same as WT, except for k_23_ = 800 M^–2^s^–1^, k_25_ = 50 s^–1^, k_34_ = 0 s^–1^, and k_43_ = 0 s^–1^. Again, k_54_ = 140 M^–1^s^–1^ and k_52_ = 0.2 s^–1^ were determined by microscopic reversibility. u = FV/RT, α' = 0.3, δ = 0.7. α" = 0.

The steady-state and presteady-state kinetics of WT SGLT1 has been described by a nonrapid-equilibrium, ordered, 6-state model.^19^ The protein alternatively faces the external and internal membrane surfaces (Figure 9). Two external Na^+^ ions bind to the empty transporter C1 to form C2 before glucose. After sugar binding, the fully-loaded transporter (C3) undergoes an isomerization, resulting in the substrate-binding sites facing the cytoplasm (C4), where glucose is released (C5) before Na^+^ (C6). The model includes a Na^+^ leak (C2 ↔ C5) in the absence of glucose. In WT SGLT1, the steady state distribution of the 6-states in the absence of sugar and the presence of 100 mM Na^+^ is 95% in C2 at −150 mV and 100% in C6 at +50 mV. Rapid jumps in membrane voltage between −150 and +50 mV produce transient charge movements (Q) with a V_0.5_ of −50 mV. The addition of external glucose redistributes the WT state distribution to 70% in C5 and 25% in C3 at −150 mV and 100% in C6 at +50 mV. This eliminated charge transfer for voltage jumps between −150 and +50 mV.

For mutant Q457R, translocation of sugar across the membrane from state 3 to state 4 is blocked (k_34_ = k_43_ = 0). The lower affinity for sugar and phlorizin binding to C2Na2 is accounted for by reducing the ratios k_23_/k_32_ and k_27_/k_72_. In the absence of external glucose, there is an increase in Na^+^-leak due to increases in the rate-constants (k_25_, k_52_) for the transition between C2 and C5. Subsequently, the state distribution at −150 mV changes to 45% C2 and 50% C5 at −150 mV, and 100% in C6 at +50 mV. The increase in Na^+^ leak (k_25_) reduces the charge transfer by ∼20% when membrane voltage is rapidly jumped from −150 to + 50 mV. On addition of external glucose, occupancy in C3 is increased to 90% at the expense of C5 at −150 mV and 100% in C6 at +50 mV. Thus, when voltage is jumped between −150 and +50 mV, there is a shift of V_0.5_ to positive values and an increase in Q_max_ (Figure 8B).

### Molecular Basis for Q457R Transport Defect

**Figure 10.**
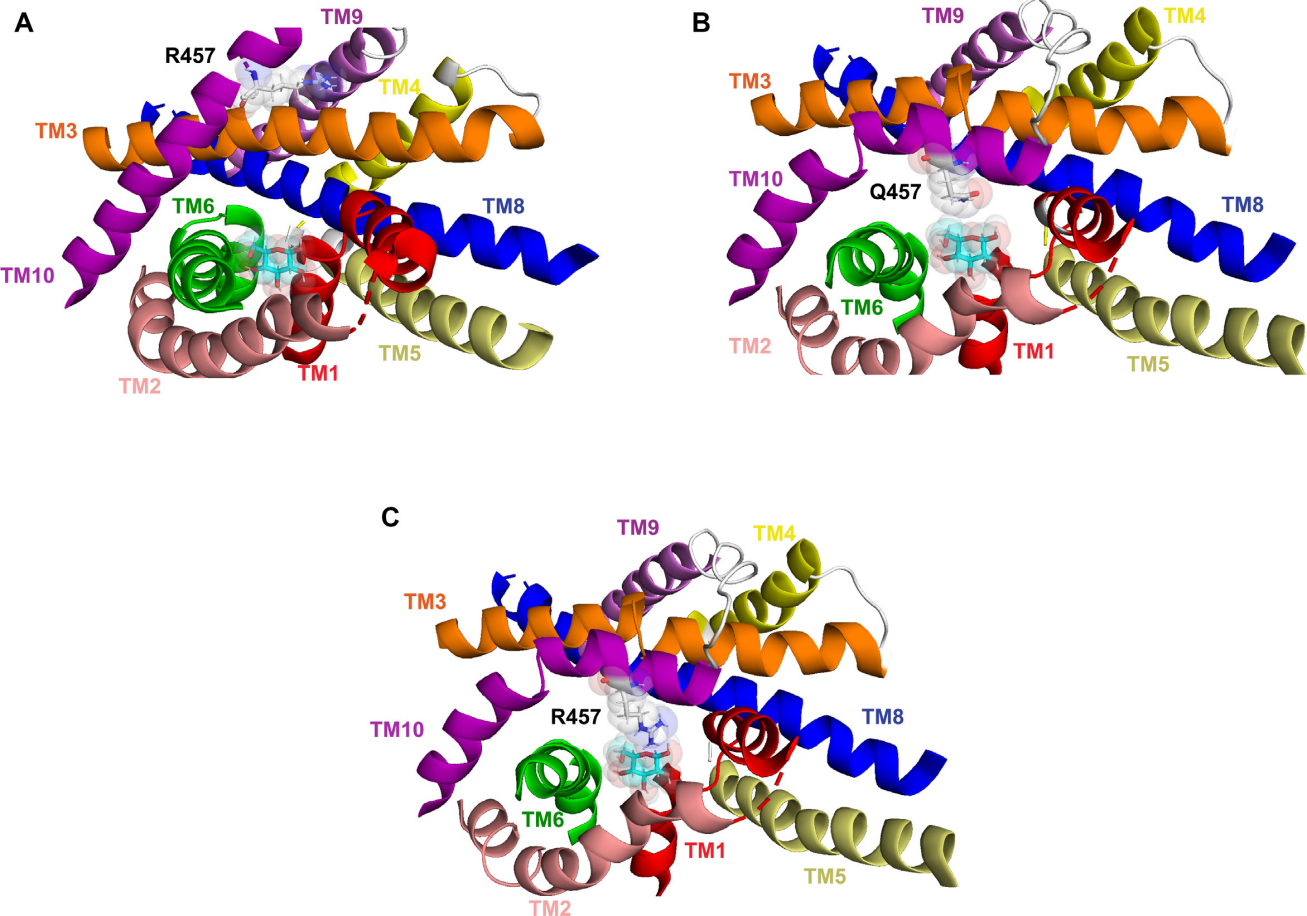
Molecular model showing sugar-binding site of WT hSGLT1 and mutant Q457R. The molecular models of the outward and inward-facing conformations of hSGLT1 were based on the crystal structures of the sodium sialic acid symporter SiaT from *Proteus mirabilis* (PDB ID 5NV9) and the sodium glucose cotransporter vSGLT from *Vibrio parahaemolyticus* (PDB ID 3DH4).^34^ Computational and experimental approaches were used to validate the homology models. A sugar-binding site in outward open conformation. The sugar coordinating residues Q457, H83, Y290, N78, and E102 are not shown. Note that Q457R does not interact with sugar in this conformation. B. The sugar-binding site in the inward conformation. Note that the inward tilt of TM10 positions Q457 to interact with the pyranose oxygen and the C#6 –OH of glucose. C. Sugar binding site for mutant Q457R in the inward conformation. Note that in this conformation the side-chain of Arg457 encounters steric hindrance to binding with glucose.

What is the molecular basis for the Q457R transport defect? Clues emerge from functional studies of other Q457 mutants expressed in oocytes: First, the Q457C-mutant, unlike Q457R, transports αMDG with a K_0.5_ of 13 mM compared to 0.5 mM for WT, and a Na^+^/glucose coupling coefficient of 6 vs. 2 for WT.^31^ The latter reflects a large Na^+^-leak through Q457C. Phlorizin blocked transport with a K_i_ 30-fold higher than WT; second, chemical alkylation of Q457C with MTSEA^+^ (2-aminoethyl methanethiosulfonate hydrobromide), MTSHE^0^ (2-hydroxyethyl methanethiosulfonate), and TMR6M (tetramethylrhodamine-6-maleimide), but not MeMTS^0^ (methyl methanethiosulfonate) or iodoacetamide, blocks transport but not sugar binding^32–34^; and third, Q457E supports Na^+^/glucose cotransport with an αMDG K_0.5_ of 2.8 mM.^33^ These data suggest that the positively-charged and bulky arginine at position 457 blocks the translocation of glucose bound from the outward-facing conformation to the inward-facing conformation. Finally, inspection of structural models of human SGLT1 indicates that glucose is not bonded with Q457 on the outer end of helix 10 in the outward-open conformation but is in the inward-facing conformation with H-bonding to the pyranose oxygen and the C6 hydroxyl group.^34^^,^^35^ This is facilitated by the inward movement of the outer ends of transmembrane helix 10 (TM10) toward the glucose binding site, bringing Q457 within range for H-bonding.^14^^,^^34^ However, due to the bulky arginine, there is an obstruction to the inward movement of Q457R on helix 10 towards the sugar-binding site (Figure 10). We propose the bulky positively-charged arginine at position 457 prohibits the inward tilt of TM10, thereby blocking glucose translocation.

## Summary

We have increased the number of GGM cases assigned to an extensive pedigree in Västerbotten County, Sweden, traced to the late 17^th^ century to 21, and identified the mutation Q457R in the *SGLT1* (*SLC5A1*) gene responsible for malabsorption. In a heterologous expression system, *X. laevis* oocytes, we have found that the mutant protein is inserted into the plasma. In a duodenal biopsy from a GGM patient the mutant protein is in the brush border membrane of enterocytes. Among GGM patients, the insertion of Q457R protein into the plasma membrane is unique as other mutations result in trafficking defects between the ER and the membrane. The defect in glucose transport by Q457R is due to a failure in the translocation of bound glucose across the membrane. This is likely caused by the positive charge on the bulky arginine side chain restricting a key conformation change in the outer end of transmembrane helix 10, preventing occlusion of the glucose binding site. In conclusion, studies of this isolated pedigree of GGM patients confirm the autosomal recessive mode of inheritance and provides unique insight into the molecular mechanism of glucose transport by SGLT1.

## Data Availability

The data underlying this article will be shared on reasonable request to the corresponding authors.

## Notes

Designated for communications with Editorial Office

**Current Addresses**
